# A transmission electron microscopy investigation suggests that telocytes, skeletal muscles, myoblasts, and stem cells in common carp (*Cyprinus carpio*) respond to salinity challenges

**DOI:** 10.1186/s12917-024-03916-0

**Published:** 2024-02-24

**Authors:** Diaa Massoud, Hanan H Abd-Elhafeez, Walaa F. A. Emeish, Maged Fouda, Fayez Shaldoum, Barakat M. Alrashdi, Mervat Hassan, Soha A Soliman

**Affiliations:** 1 Department of Biology, College of Science, Jouf University, Sakaka, Al-Jouf, 72341 Saudi Arabia; 2 Department of Cell and Tissues, Faculty of Veterinary Medicine, Assiut University, Assiut, 71526 Egypt; 3 Department of Fish Diseases, Faculty of Veterinary Medicine, South Valley University, Qena, 83523 Egypt; 4 Department of Theriogenology, Faculty of Veterinary Medicine, New Valley University, El Kharga, Egypt; 5 Department of Histology, Faculty of Veterinary Medicine, Qena, Egypt

**Keywords:** Telocytes, Salinity, Stem cells, Myoblasts, ***Cyprinus carpio***, Skeletal muscles

## Abstract

**Background:**

Telocytes are modified interstitial cells that communicate with other types of cells, including stem cells. Stemness properties render them more susceptible to environmental conditions. The current morphological investigation examined the reactions of telocytes to salt stress in relation to stem cells and myoblasts. The common carp are subjected to salinity levels of 0.2, 6, and 10 ppt. The gill samples were preserved and prepared for TEM.

**Results:**

The present study observed that telocytes undergo morphological change and exhibit enhanced secretory activities in response to changes in salinity. TEM can identify typical telocytes. This research gives evidence for the communication of telocytes with stem cells, myoblasts, and skeletal muscles. Telocytes surround stem cells. Telopodes made planar contact with the cell membrane of the stem cell. Telocytes and their telopodes surrounded the skeletal myoblast. These findings show that telocytes may act as nurse cells for skeletal stem cells and myoblasts, which undergo fibrillogenesis. Not only telocytes undergo morphological alternations, but also skeletal muscles become hypertrophied, which receive telocyte secretory vesicles in intercellular compartments.

**Conclusion:**

In conclusion, the activation of telocytes is what causes stress adaptation. They might act as important players in intercellular communication between cells. It is also possible that reciprocal interaction occurs between telocytes and other cells to adapt to changing environmental conditions.

## Introduction

A telocyte (TC) is a modified interstitial cell that communicates with different types of cells and participates in a wide range of biological roles in various tissues and organs [1]. . Telocytes are characterized by their cell prolongations, or “telopodes,” derived from the cell body. The size of the cell body and telopodes, as well as the detection of telopodial emergence, confirm the ultrastructural morphology of telocytes [2]. . Telopodes form a complex labyrinthine network and provide a cellular communication system. They have narrow segments, podomers, and interval expansions, as well as podoms that are densely packed with mitochondria, endoplasmic reticulum, and caveolae [3]. Paracrine signaling and cellular interaction are two more ways in which telocytes influence other cells. Interactions between cells can be homocellular or heterocellular. Heterocellular connections (including minute junctions, point contacts, nanocontacts, and planar contacts) between telocytes and other cells, and cell-to-cell contact at the intermembrane distance, enable macromolecule interaction [4]. . Various types of cell contact are specified, including adherence (both puncta adherents minimum, processes adherents, and manubria adherents), gap junction, and direct apposition of adjoining telocytes’ cell membranes. A gap junction is essential for the transfer of signals between cells [4, 5]. . To other cells, telocytes deliver microvesicles and macromolecules like RNAs and proteins. Multiple kinds of extracellular vesicles are secreted by these cells, including exosomes, ectosomes, and multivesicular vesicles [3, 6, 7].

Regarding protein analysis, various functions have been described for telocytes. Further biological analysis identified 38 proteins associated with telocytes. These proteins are involved in intercellular communication through the transit of vesicles and the development of cellular structures. The majority of these proteins are cytoskeletal proteins and oxidoreductases [8]. . Gene expression analysis of chromosome 4 in murine pulmonary telocytes identify 17 up-regulated genes and 56 down-regulated genes. The authors concluded that gene function is related to cellular signaling, regulation of tissue inflammation, cell expansion, and movement [9]. The gene profiles on chromosomes 2 and 3 of pulmonary telocytes differentiate between 26 and 80 genes in chromosome 2 and 13 or 59 genes in chromosome 3. The presence of Myl9 in chromosome 2 of TCs indicates the involvement of telocytes in tissue and organ damage as well as the aging process. The decrease in Pltp expression suggests that telocytes have an anti-inflammatory effect. Tumor promotion was observed in chromosome 3 due to the presence of Sh3glb1, Tm4sf1, or Csf1 expression. The gene Pde5, which is commonly down-regulated in pulmonary telocytes, plays a crucial role in the development of pulmonary fibrosis and other acute and chronic interstitial lung disorders [10]. . The role of telocytes in cardiac embryogenesis has been investigated in mice. A three-dimensional organized network between myocardial precursors implies a telocyte role in myocardial arrangement during heart development [11]; Pulmonary telocytes express angiogenic-associated proteins, including Nidogen, Collagen type IV, and Tissue Inhibitor of Metalloproteinase 3 (TIMP3), so telocytes are involved in angiogenesis and tissue remodeling [12]. Multiple studies have investigated the importance of telocytes in the process of regenerating various organs, including the heart, lung, skeletal muscle, skin, meninges and choroid plexus, eye, liver, uterus, and urinary system [13]. .

However, stem cells have a fundamental role in tissue regeneration. Two general features have been used to define stem cells: their capacity for self-renewal and differentiation. Stem cell niche is a defined tissue microenvironment that modulates the retaining of stemness state, proliferation, and differentiation of stem cells. The stem cell niche contains a high concentration of growth factors, miRNAs, and tiny vesicles, which are transported through autocrine and paracrine mechanisms. Differentiation and lineage specification of stem cells depends on the molecular composition of the ECM (extracellular matrix) [14, 15].

Regarding cellular contact between telocytes and stem cells, there is speculation that telocytes nurse cardiomyocyte progenitors during differentiation [16]. The cellular interaction between telocytes and both cardiac and hematopoietic stem cells has been studied by using fluorescent cells and extracellular vesicles labelled with calcein and Cy5-miR-21 oligos to detect the pathway of paracrine signalling. Telocyte-stem cell interaction is mediated through microRNA-rich secretory vesicles [17].

Being undifferentiated, stem cells are susceptible to environmental factors. Environmental stressors drive changes in stem cell function and fate. Cellular stress may promote quiescence, active apoptotic death programmed, induce genetic alterations, and acquire oncogenic mutations. Cell stress occurs upon exposure to specific intrinsic or extrinsic factors. Intrinsic stress result from of the normal metabolic processes that liberate noxious waste products and reactive metabolites. Extrinsic environmental factors alter the biological activities of cells [18]. Several studies have explored the regenerative tendency of stem cells subjected to stress [19, 20]. Scientists have looked into using mesenchymal stem cells to treat changes in the hippocampus CA1 (Cornu Ammonis1) region of Wistar rats caused by cold stress. Administration of bone marrow mesenchymal stem cells through intravenous injection enhances the mitigating effect on cold-induced stress. The bone marrow mesenchymal stem cell-treated group exhibited an augmentation in the quantity of nerve cells. The authors’ findings indicate that the intravenous administration of bone marrow mesenchymal stem cells resulted in the generation of new neurons in the hippocampal CA1 region [19]. . Hypoxic stress promotes the process of stem cell de-differentiation, leading to the acquisition of embryonic stem cell characteristics, as well as facilitating their differentiation and proliferation [21]. .

The purpose of this research is to examine telocytes in the gills of the common carp (family Cyprinidae, Common Carp Cyprinus carpio), which is considered an important and significant aquaculture species and is used as an experimental model in aquatic species [22, 23]. This study involved changing the exterior environment, associated with a wide range of cellular adaptive responses. Furthermore, its objective is to investigate the correlation between salinity-induced morphological alterations of telocytes and their associations with stem cells, muscular progenitor cells, and skeletal muscles within the gills of common carp.

## Materials and methods

### Ethical declaration

The protocols of this study were approved by the National Ethics Committee of South Valley University, Egypt (Approval NO. 8a/13.12.2020). The experiment was conducted in South Valley University in accordance with the ARRIVE (Animals in Research: Reporting In Vivo [24] Experiments) criteria. All methods were performed in accordance with the relevant guidelines and regulations.

### Source and transportation of fish

The common carp, scientifically known as *Cyprinus carpio*, was introduced into the aquariums from a commercial fish farm in the governorate of El-Dakahlea in enormous water containers. During transport, the oxygen concentration was maintained at 5 mg/l, the temperature was maintained at 23 °C ± 3, and the pH range was maintained at 7.2–7.5.

### Fish acclimation

The fingerling fish appeared to be one month old and had a healthy body measurement of approximately 7 ± 2 cm. Its total mass was 10 ± 2 g. Following the collection process, the fish were transported to the wet laboratory at the Faculty of Veterinary Medicine in Qena, Egypt, which is part of South Valley University. The fish spent three weeks in a controlled environment before the trials began, They adjusted to water with a salinity of 0.2 parts per thousand during that time. Their daily protein intake constituted 3% of their body weight and was distributed evenly between two meals consisting of a commercial floating powdered feed with a protein content of 45%.

### Aquaria

The initial method for keeping fish for bioassays, as outlined in reference [25] Utilized a recirculating system within porcelain tanks measuring 260 × 65 × 70 cm. The experimental setup consisted of 60 × 30 × 40 cm fiberglass aquariums. The pH level was maintained within the range of 7.2 to 7.5. The water temperature was kept at 23 °C ± 3. The content of dissolved oxygen was above 5 mg/l.

### Salinity exposure

Electrical conductivity was used to measure the salinity of the water [20]. Thirty-six settled in, looking fine. The experimental groups consisted of common carp, *C. carpio*, weighing 9 and 11 g. The experimental groups consisted of 12 fiberglass tanks with dimensions of 60 × 30 × 40 cm. Each group had 9 fish, and each salinity group had 3 repetitions. Three distinct groups were exposed to salinities ranging from 6 to 10 parts per thousand. Every two days, the tanks were emptied, and 2 g/L of NaCl was added to each group to raise the salinity to the appropriate level. The fourth set of animals in the study were raised in a control group, where they were exposed to naturally occurring water. This water was dechlorinated tap water with a salt concentration of 0.2 parts per thousand. Before sample collections, fish were given at least two weeks to adjust to the different salinities.

### Histological investigation

### Collecting fish samples for ultrastructural analysis

Fish that were in good health were used to collect the samples. For every salinity level, nine fish were beheaded. The gill arches and filaments on both sides were meticulously removed. The samples were fixed for transmission electron microscopy using a solution consisting of 10 millilitres of 5% glutaraldehyde and 90 millilitres of 0.1 M Na-phosphate buffered formalin.

### Preparation of resin-embedded specimens for cutting into semi-thin and ultra-thin sections

Small portions of fixed gill filament and arch samples were cut into pieces. The specimens underwent four 15-minute treatments with 0.1 M sodium phosphate buffer, followed by a two-hour post-fixation in 1% osmic acid in 0.1 M Na-phosphate buffer at 4 °C. The osmicated samples were submerged in a 0.1 M phosphate buffer (pH 7.2) and washed three times for 20 min each. Ten minutes of dehydration was conducted with progressively higher concentrations of acetone (70, 80, 90, and 100%). Following the dehydration process, the samples were submerged in a solution consisting of resin and acetone for a duration of one day. Subsequently, they were immersed in a solution containing half resin for an additional day, and lastly soaked in pure resin for a period of three days. The resin was made by thoroughly mixing 10 g of ERL, 6 g of DER, 26 g of NSA, and 0.3 g of DMAE using a shaker. After three days of immersion in the resin at 60 °C, the specimens were removed. The polymerized materials were cut into semi-thin sections and dyed with toluidine blue using an ultramicrotome called Ultracut E, manufactured by Reichert-Leica in Germany [26]. .

The Assiut University JEOL100CX II transmission electron microscope (TEM) was employed to analyze the (70 nm) sections obtained from ultrathin sections stained with uranyl acetate and lead citrate.

### Digital coloring of transmission electron microscopy images

The transmission electron microscopy pictures was colored using the Photo Filter 6.3.2 software. To modify the color degree, you must apply color to images by using the color tool to mark the specific cells [27–29].

## Results

**Fig. 1:**
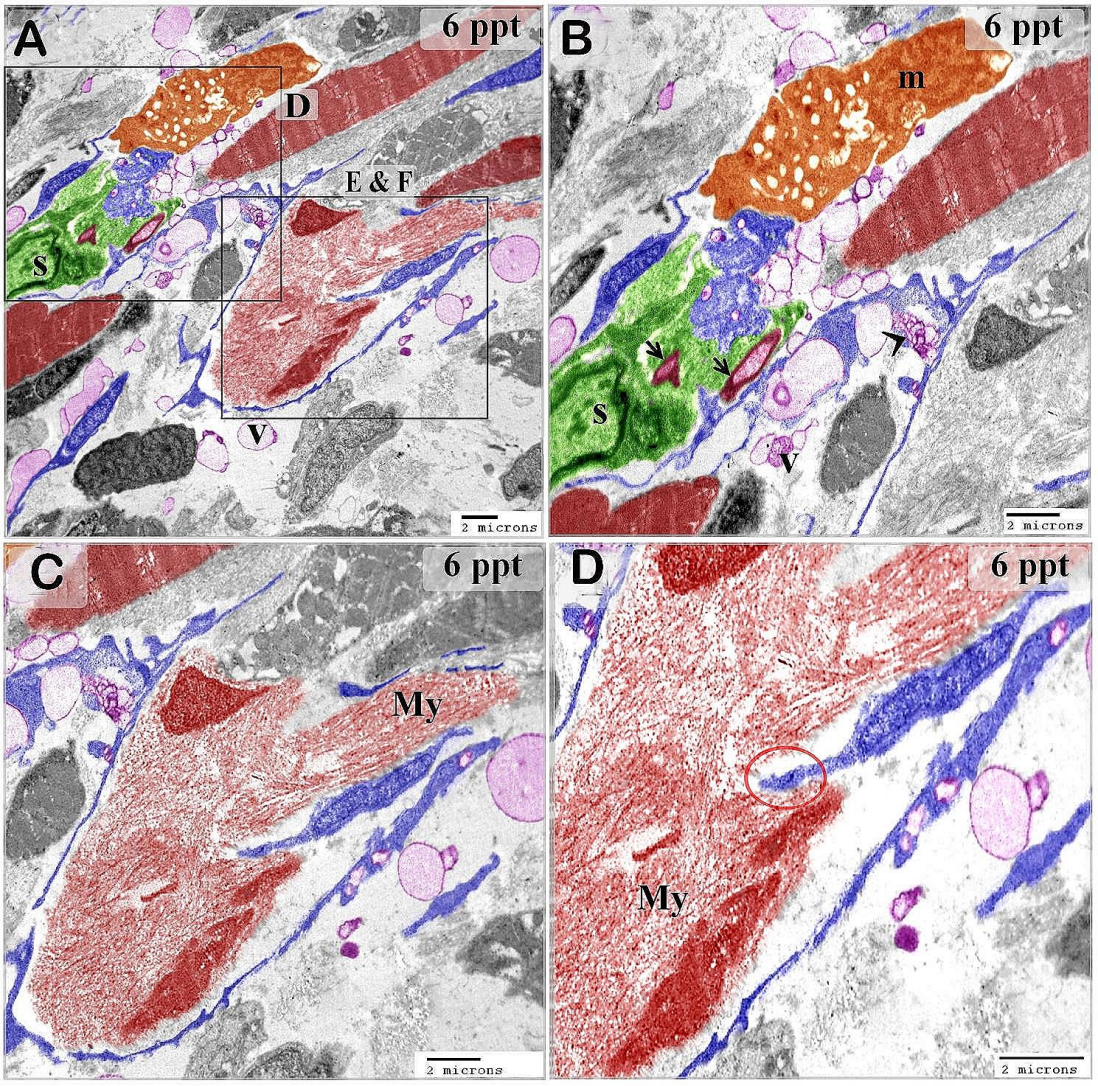
Telocytes relation with skeletal myoblast. Colored ultra-thin sections in gill arches treated samples with a 6 ppt level of salinity. **A-D**: Telocytes surrounded the myoblast which contained ill-organized myofibrils (My). Telopodes were connected to nerve fibers and formed multipoint contact with skeletal muscles. Note nerve fiber (arrow), secretory vesicles (V), and multivesicular body (arrowhead). Telocytes established contact with different types of stromal, epithelial, stem cells (green color), and skeletal muscles. Large number of secretory vesicles were excreted close to the muscular fibers in samples treated with a 6 ppt concentration of salinity

**Fig. 2:**
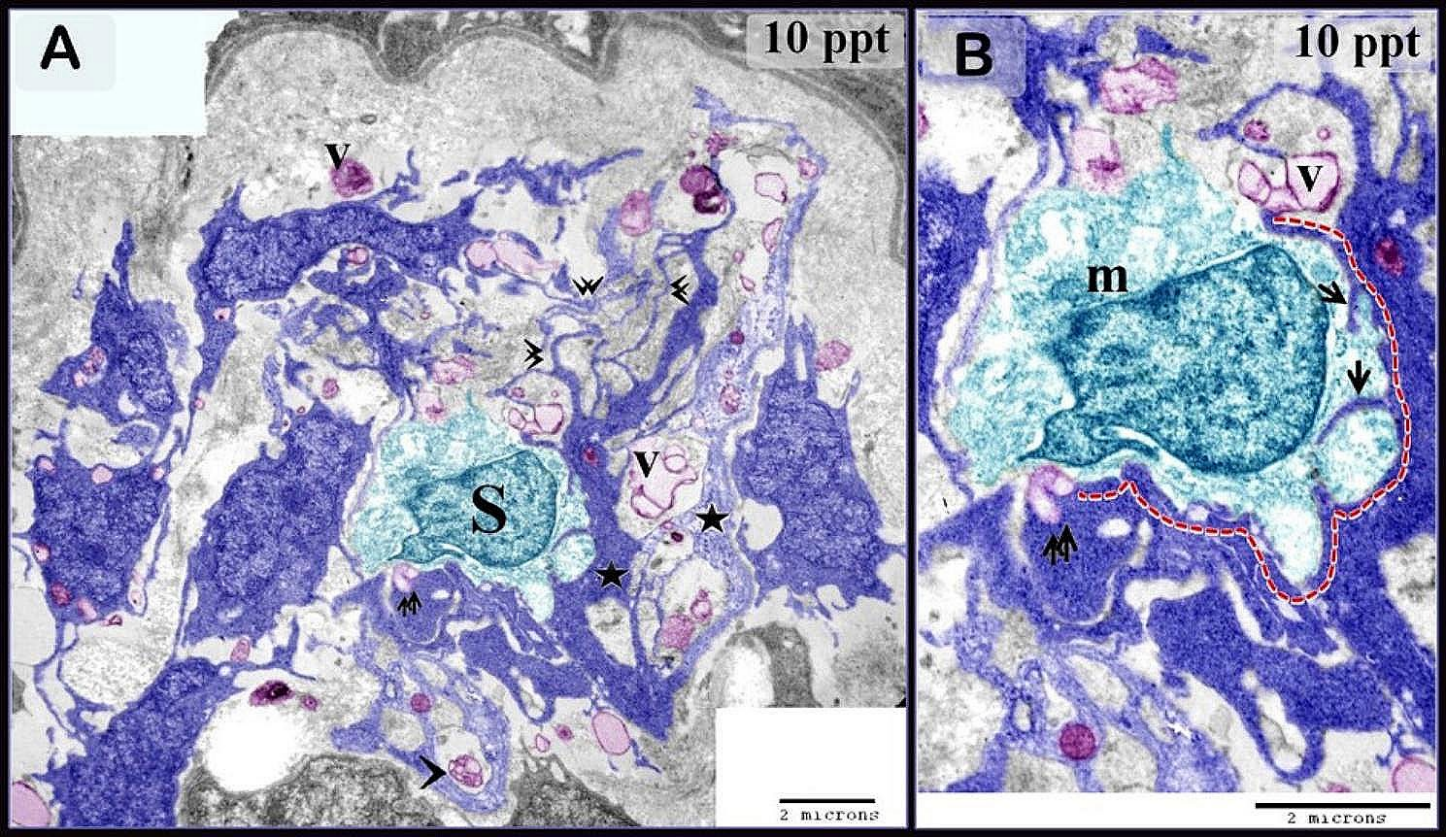
Telocytes relation with stem cells. Colored ultra-thin sections in gill arches treated samples with 10 ppt (**A, B**) level of salinity **A, B**: several telocytes surrounding stem cells (S), which are characterized by a high nuclear-cytoplasmic ratio and contain mitochondria (m). Telopodes established a planar contact with stem cells (dashed line). Telopodes formed an extensive network (double arrowheads). They secreted vesicles (V) and a multivesicular body (arrowhead). Several telopodes interdigitate with the stem cells (arrows). note secretory vesicles attached to stem cells (double arrow). Some telopodes were thickened (asterisk)

This research investigated the response of telocytes to varying levels of hypertonic circumstances and their association with stem cells, muscle progenitor cells, and skeletal muscles in the gills of common carp. Telocytes were identified by TEM in the gill arches of the common carp, which had typical characteristic features. Telocytes possess a cell body that is accompanied by thin cellular extensions known as telopodes. Telopodes consisted of podoms and podomers (Fig. 1A, C). They shed secretory vesicles, exosomes, and multivesicular vesicles (Fig. 2A).

**Fig. 3:**
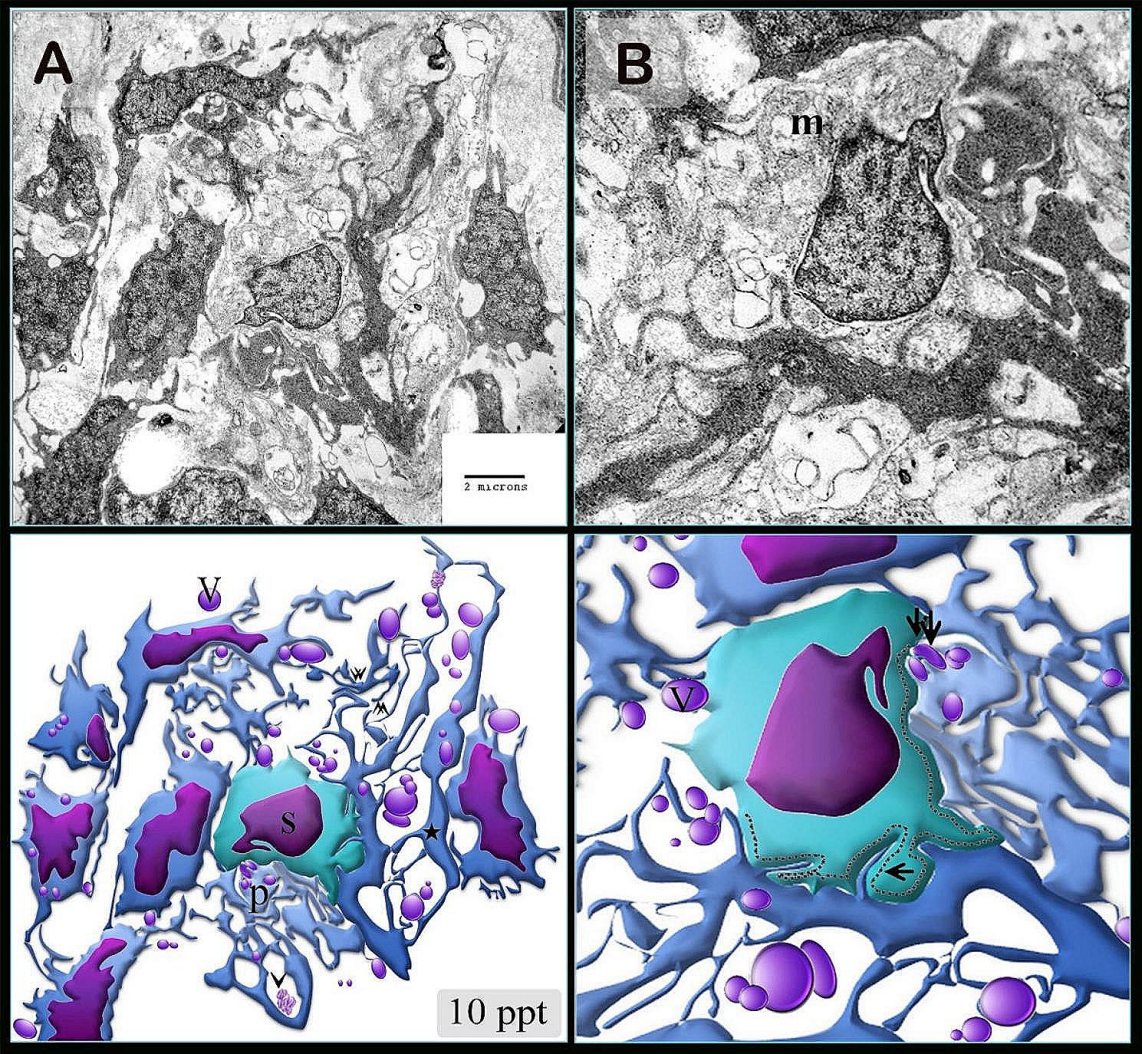
Figure 2 original transmission electron microscopy beside the figure’s representative drawing

**Fig. 4:**
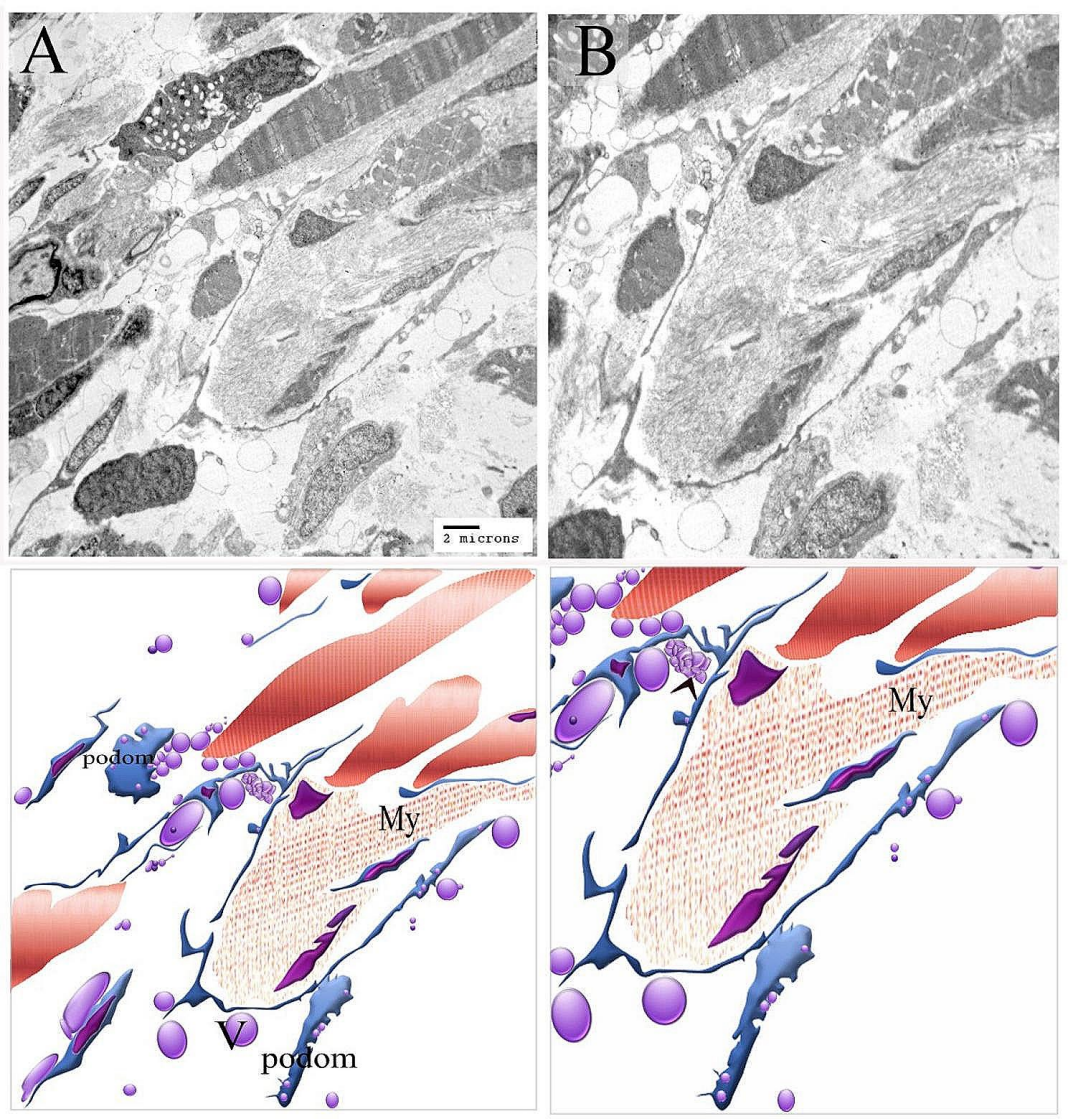
Figure 1’s original transmission electron microscopy beside the figure’s representative drawing

Figures 3 and 4 illustrate the original transmission electron microscopy (TEM) images alongside the corresponding schematic illustrations presented in Figs. 2 and 1, respectively.

**Fig. 5:**
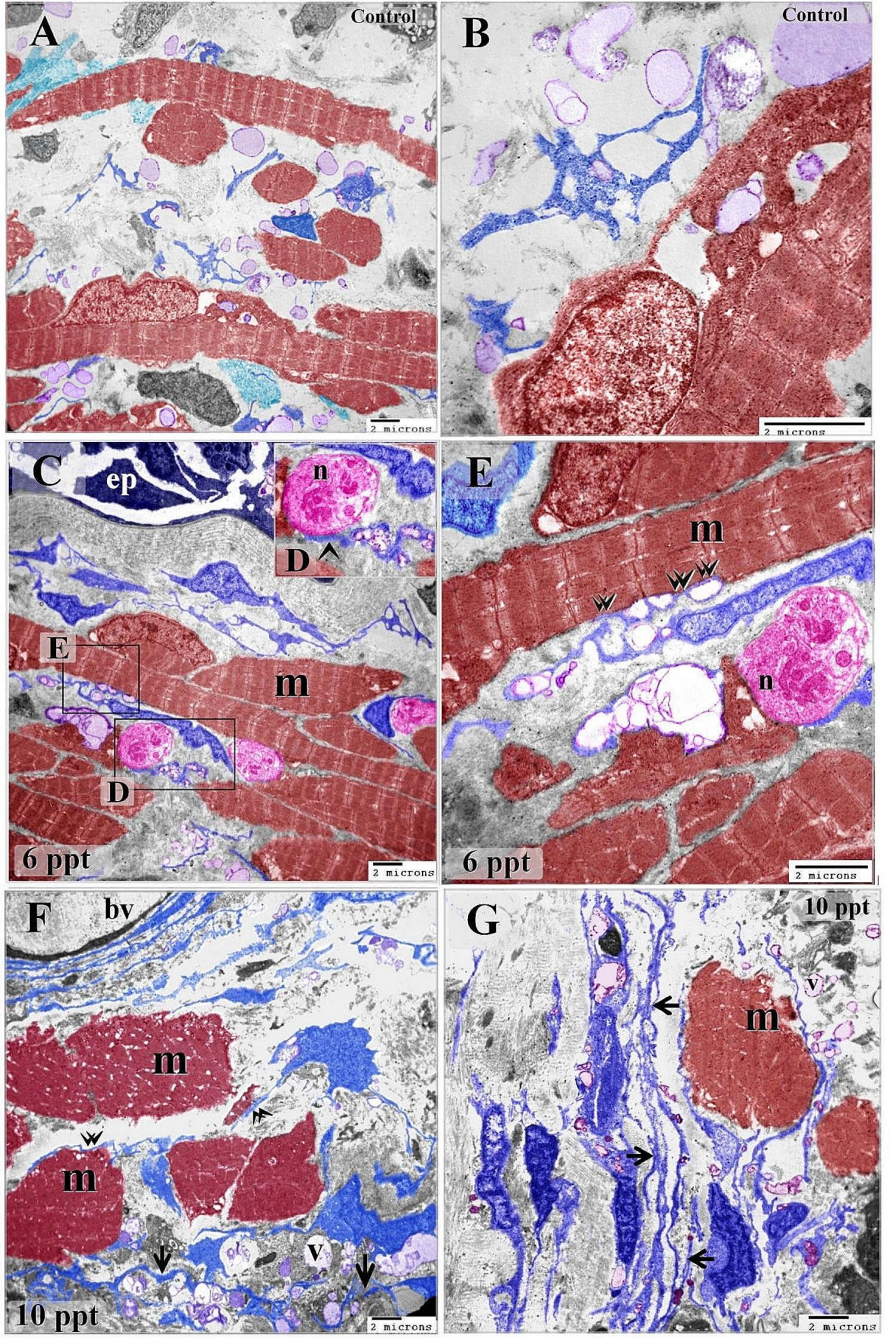
Skeletal muscle fibers undergo hypertrophy in response to salinity. Colored ultra-thin sections in gill arches control (**A**) and treated samples with 6 ppt (**A-C**),10 ppt (**D, E**) level of salinity. A, B: in control samples, telopode (T) adjacent to skeletal muscle fiber which received telocytes secretory vesicles. Note the secretory vesicles (arrows) located in intracellular compartments (**C**) in the muscle cell. Note extracellular secretory vesicles (V). **C, D, E**: In 6ppt treated samples, telocytes (blue color) formed a network between muscle cells (red colored). Telocytes established multi-point contact (double arrows) with skeletal muscle fiber (m). Telopode formed a direct contact with the nerve fiber (n). Note epithelium (ep). **F, G**: In 10 ppt treated samples, telocytes established direct contact (double arrowheads) with skeletal muscle fibers which increased in diameter (m). Note telopodes organized an extensive network and acquired a corrugated appearance (arrows). Note blood vessels (bv) and secretory vesicles (V)

The gill-arch stroma comprised stem cells with a high nuclear-cytoplasmic ratio and mitochondria. Telopodes of numerous telocytes encircled the stem cells, forming a large network that partially enveloped the stem cells. Telopodes made a planar contact along the cell membrane of the stem cell; telopodes produced interdigitated cytoplasmic processes extending towards the stem cell. (Fig. 2A, B). Telocytes and telopodes encircled the skeletal myoblast, which organized the myofibrils. Gradual expansion of myoblasts with increasing salinity levels (Fig. 1A, B, C, D, and 5A–C).

**Fig. 6:**
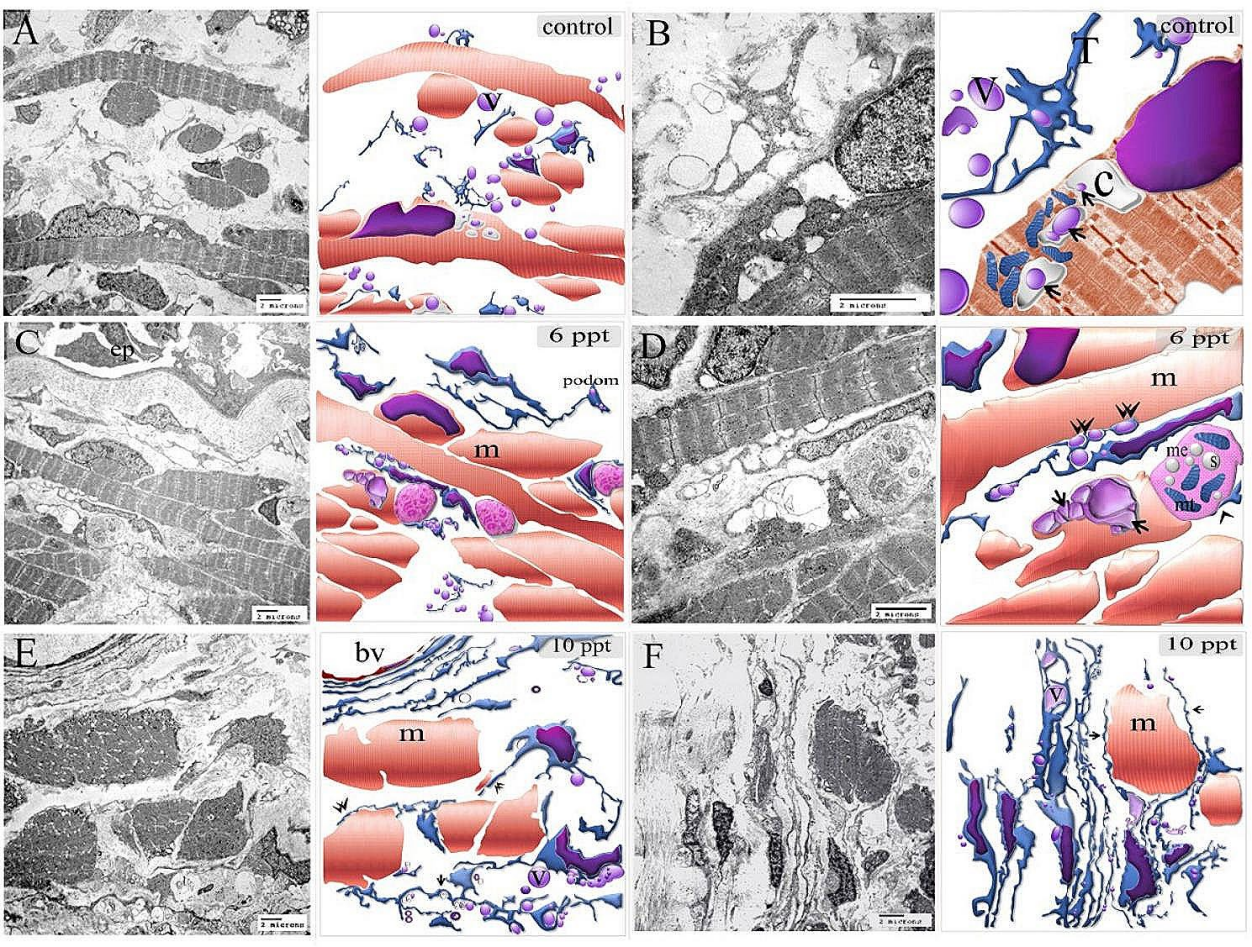
Figure 5’s original transmission electron microscopy beside the figure’s representative drawing

Telocytes make connections with skeletal muscles. Telopodes were linked to nerve fibers (Fig. 5C-E) and established either single-point contact (Fig. 5F) or multi-point contact (Fig. 5C, E) with skeletal muscles. Skeletal muscle fibers receive telocyte secretory vesicles. The secretory vesicles are located in intracellular compartments in the muscle cell (Fig. 5A, B). A large number of secretory vesicles were released proximal to the muscle fibers in samples treated with a salt concentration of 6 ppt (Fig. 5C-E). An immense network of telopods was discovered, and telocytes shed more secretory vesicles. Telopodes developed a corrugated look. In comparison to the control samples, skeletal muscle fibers experience hypertrophy after attaining a salt concentration of 10 ppt (Fig. 5F, G). Figure 6 illustrate the original transmission electron microscopy (TEM) images alongside the corresponding schematic illustrations presented in Fig. 5, respectively.

**Fig. 7:**
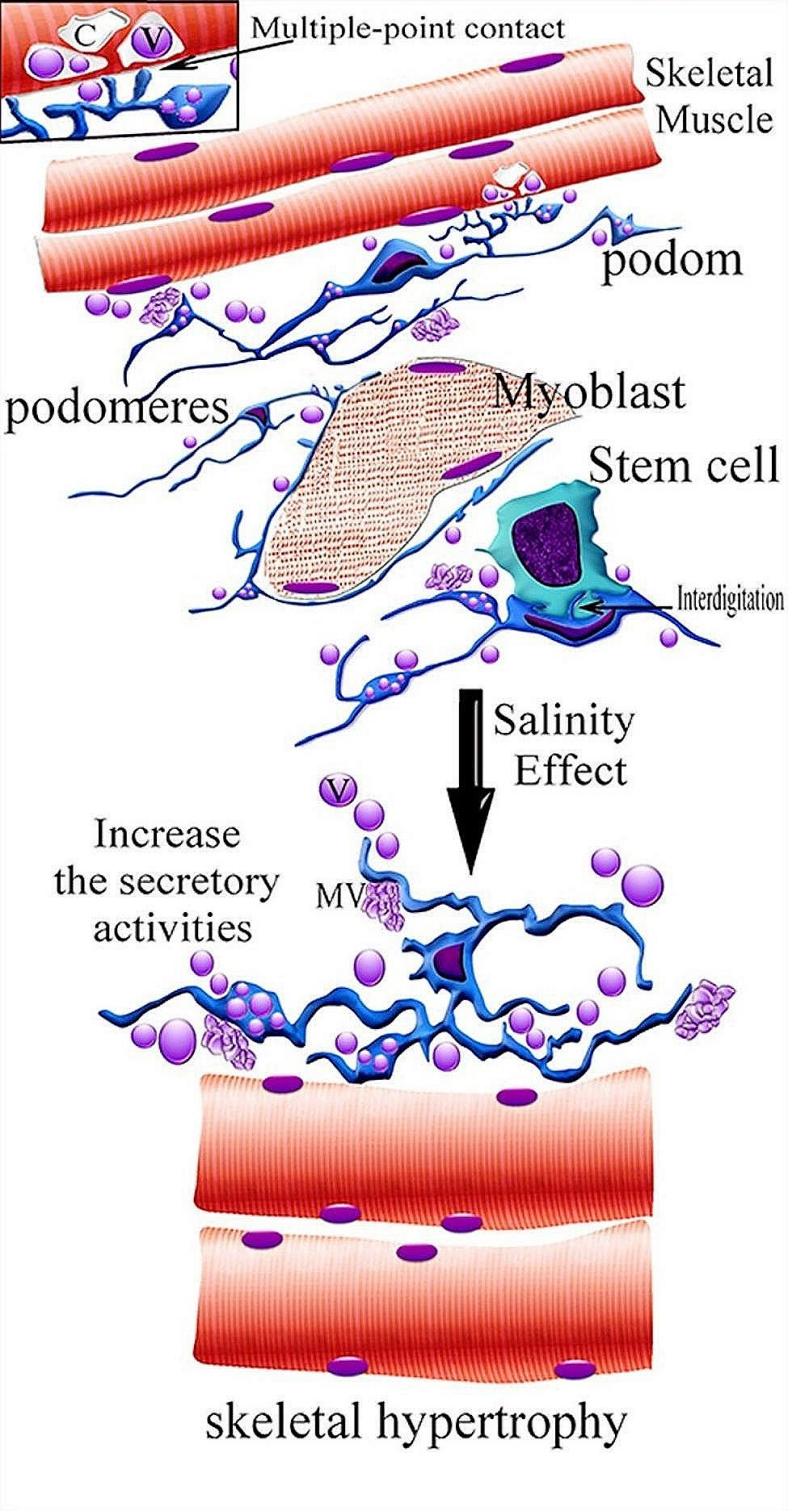
An illustration shows the relations of telocytes with skeletal muscles, myoblasts, and stem cells and the effect of salinity on telocytes and skeletal muscles. Telocytes have telopodes which consisted of podoms and podomeres. Note multivesicular body (MV), the secretory vesicles (V) were phagocytosed in intracellular compartments (c). Telocytes formed multiple-point contact with skeletal muscle, planer contact was formed between telocytes and stem cells. Note interdigitated cytoplasmic projections or cytonemes of telocytes projected to stem cells. In high salinity levels, telocytes increased the secretory activities, telopodes acquired a corrugated appearance and skeletal muscles undergo hypertrophy

An illustration shows the relations of telocytes with skeletal muscles, myoblasts, and stem cells and the effect of salinity on telocytes and skeletal muscles (Fig. 7).

## Discussion

The current research aimed to morphologically examine telocyte responses to salt stress and their interaction with stem cells and skeletal myoblasts. We identified telocytes using ultrathin sections. Common carp gills had telocytes with telopodes and podoms. Telopodes made homo- and heterocellular connections. Heterocellular contact was formed between stem cells, skeletal myoblasts, and muscle fibers. Telocytes in fish species were morphologically similar to those in mammalian, avian, and amphibian species [30–32]. . There are two hypotheses for telocytes. Some studies hypothesized that communities of telocytes expressing stem cell markers existed, and second hypotheses involving morphological criteria are also presented. Regarding the first hypothesis, some researchers hypothesized that populations of telocytes expressing stem cell markers, particularly c-kit/CD117 and vimentin, were deemed a subset of mesenchymal stem cells. While other groups express CD34 and vimentin [31]. . CD34 and PDGFR are considered common markers for the identification of telocytes. However, variable immunohistochemical affinities have been recognized for telocytes in the same organ and are hence termed telocyte subtypes. PDGFR-positive or SMA-negative sub-epithelial telocytes, PDGFR-positive or SMA-positive deeper large telocytes, and CD34-positive or SMA-negative submucosal telocytes have been detected in the human urinary bladder [33]. In the human mammary gland, interstitial cells morphologically identical to telocytes are distinguished using CD34+/CD10±/c-kit-/vimentin+. The authors speculated that these cells are likely to act as mammary stem cells [34]. In the human heart, subendocardial interstitial cells exhibit neuron-specific markers, partially enolase, neural stem cell markers, nestin, and endothelial cell-specific markers, CD146. The authors concluded that subendocardial interstitial cells could be an endocardial stem niche that may include telocyte-like cardiac progenitors [35]. Regarding the second hypothesis, the telocyte subtypes are characterized in terms of morphological criteria. The first subtype consists of a smaller oval body and at least two long, thin varicose processes. The second kind is larger in size, with prominent rough endoplasmic reticulum cisternae and an evident Golgi apparatus. Large telocytes are termed hybrid telocytes, which form plaques identical to fibronexus junctions in myofibroblasts in the human bladder [33]. Based on immunophenotypic characteristics, it is also believed that telocytes represent a differential stage of Interstitial Cells of Cajal (ICC) An in vitro study explored the immunohistochemical phenotype of interstitial cells and identified kit-negative and CD34-positive cells. The authors suggested these cells are ICC progenitors, undergo differentiation, and acquire the C-Kit surface [36, 37]. Telocyte subtypes are regarded stem cells that differentiate into diverse types of cells, such as Cajal’s interstitial cells, resident myofibroblasts, and fibroblasts, brain tissue [33, 38]. . Gene expression analysis of telocytes and mesenchymal cells distinguished differences in cellular activities. Telocyte chromosome 1 contains approximately 25% up-regulated and 70% down-regulated genes. Fourteen genes found on telocyte chromosome 1, including (Ralb, Igsf8, Sdpr, Csrp1, Uck2, Rab3gap2, Arpc2, Nav1, Psmd1, Tagln2, Tpp2, Capn2, Fhl2, Qsox1) on chromosome 1 of TCs were up-regulated (> 1 fold). According to the authors, telocytes are a different type of cell whose primary tasks include control, morphogenesis, and tissue homeostasis [39]. .

In the current work, telocytes established planer contact with stem cells. They formed interdigitated cytoplasmic processes projecting towards the stem cell. Cytoplasmic interdigitations seem to maintain a large surface area for cell contact. The cytonemes, or cytoplasmic projections, allow cell-cell contacts. Cytonemes are considered a specific form of signaling filopodia through which protein signals are dispersed at the contact site [40, 41]. Therefore, the present work supports the idea that condensed telocytes, acting as regulatory cells, offer functional assistance to stem cells. Many researchers endorse the role of telocytes in facilitating the proliferation of stem cells and progenitor cells in various organs, including the lung [42], meninges, and choroid plexus [43], heart [44, 45], liver [46], **and** skeletal muscles [47]. Many hypotheses have been suggested to elucidate the connection between telocytes and stem cells. Regarding morphological evidence, telocytes have been implicated in the regulation of cell signaling in the stem cell microenvironment, either via direct mode (cell junction) or indirect mode (paracrine signaling) [13]. Other researchers present a prospective insight into telocyte function in therapeutic regenerative medicine. The role of telocytes in skeletal muscles is not limited to regenerative capability. Their role in proliferation and angiogenesis was documented regarding in relation to the presence of the Ki67 proliferative marker, the Oct4 pluripotency marker, and the VEGF vascular proliferation marker. Skeletal muscle-derived stem cells exhibit a diverse spectrum of differentiation capacity. The skeletal muscle-derived stem cells were co-cultured with telocytes, which demonstrated the ability to differentiate into adipocytes, chondrocytes, and osteoblasts. These findings support the previous hypothesis that telocytes might have a role in tissue regeneration and repair [47]. Being telocytes that share immunoreactivity for specific markers doesn’t imply that telocytes are precursors of the cells exhibiting the specific markers. Both telocytes and some endothelial cells of the arterioles and venules are CD34-positive [48]. The proteomics profile for telocytes and endothelial cells has been estimated using the the isobaric tag for relative and absolute quantification (iTRAQ) in combination with automated 2-D nano-ESI LC-MS/MS. Telocytes exhibit an increase in the expression of 38 proteins on the 5th day and 26 proteins on the 10th day. The telocyte-associated proteins participate in intercellular communication, including vesicular trafficking proteins, cell morphogenesis, cytoskeletal proteins, and oxidoreductase enzymes. While endothelial cells express 60 up-regulated proteins, mainly cell surface glycoprotein MUC18 (15.54-fold) and von Willebrand factor (5.74-fold). The author concluded that telocytes and endothelial cells are different types of cells; each has its own biological activities. The main functions of telocytes are intercellular communication and intercellular signaling. Telocytes have the ability to inhibit oxidative damage and cellular aging, while promoting cellular renewal by limiting apoptosis [8]. . In order to learn about telocytes in the context of liver repair, after partial hepatectomy, the numbers of telocytes and hepatic precursor cells are estimated using a combination of techniques: immunofluorescence with double labeling of CD34/PDGFR-α, CD34/PDGFR-ß, and CD34/Vimentin to find telocytes; immunostaining with 5-ethynyl-2′-deoxyuridine (EdU) and Western blotting of Proliferating Cell Nuclear Antigen (PCNA) to find hepatic regenerative cells. Increases in telocytes, hepatic stem cells, and proliferating cells suggest that telocytes may facilitate hepatic regeneration through hepatocyte proliferation and activation of hepatic progenitors [49]. . The telocyte microenvironment might possibly be rich in factors that trigger stem cells to differentiate. Telocytes are thought to help potential stem and progenitor cells differentiate by creating the right conditions. Previous study has shown that pulmonary telocytes and putative stem cells can talk to each other. Telopodes created nanoparticles that connect stem cells and act as bridges [42]. .

In this research, telocytes formed a three-dimensional network in the gill arch’s stroma and were in close contact with myoblasts. The telocytes and their telopodes initiated the organization of the intracellular myofilament proteins by enclosing and coming into direct contact with the skeletal progenitor l. celThe telocytes and their telopodes surrounded the skeletal progenitor cell and made direct contact with it. This started the organization of the myofilament proteins inside the cells. Thus, telocytes may be involved in skeletal progenitor cell differentiation. The existence of telopodes is closed to skeletal muscles. Telocyte secretory vesicles were transferred to skeletal muscle cells. Several types of extracellular compartments, categorized according to size, were shed by telocytes: exosomes ranging from 30 to 100 nm, ectosomes (microvesicles) measuring about 100 and 1000 nm, and apoptotic bodies measuring 50 nm–2 μm. Biological interaction between extracellular vesicles and recipient cells occurs via endocytosis, ligand-receptor interactions, and merging to the plasma membrane. Extracellular vesicles mediate molecular transfer for intercellular communication. Extracellular vesicles convey a wide range of molecules, including receptors, bioactive lipids, proteins, and, most critically, nucleic acids like as mRNA, microRNA (miRNA), and non-coding RNAs [50]. . There are three categories of chemicals in telocyte secretomes: growth factors, chemoattractants, and cytokines/chemokines. Cardiac telocytes secrete IL-6, IL-2, IL-10, IL-13, VEGF, EGF, nitric oxide, MIP-1α and 2 (MIP-1α and MIP-2), monocyte chemoattractant protein 1 (MCP-1), and GRO-KC to promote stem cell proliferation and differentiation [45]. . The application of stem cells in tissue regeneration remains a prospective therapeutic target. Recent attempts have considered the intimate relationship between telocytes and stem cells to detect the key factors in their microenvironment and develop a new approach to regenerative medicine.

In the current study, telocytes undergo structural modification, and secretory activities were increased in response to salinity. These results suggest that telocytes are sensitive to environmental changes, which in turn affect on stem cells, myoblasts, and skeletal muscles. The results also support the idea that telocytes might act as important players in intercellular communication between cells. Moreover, it is possible that reciprocal interaction occurs between telocytes and other cells to adopt changes in environmental conditions. Previous research documented morphological alternations of telocytes exposed to the altered environment. Telocytes form additional short telopodes in an oxidative stress environment. They also exhibit a long and slender shape in N-acetyl cysteine cell culture medium [51].

Ultimately, the fish’s physiology adjusted to varying salt levels by triggering an adaptive response via cellular communication. Telocytes are a significant element of the communication system. Telocytes, through either direct contact or paracrine signaling, exhibit responsiveness to environmental changes and exert regulatory control over the functioning of stem cells and myoblasts. Therefore, telocytes have the potential to promote the formation of muscle fibers and the enlargement of skeletal muscle cells.

## Data Availability

No datasets were generated or analysed during the current study.
